# Dual Behavior of Iodine Species in Condensation of Anilines and Vinyl Ethers Affording 2-Methylquinolines

**DOI:** 10.3390/molecules21070827

**Published:** 2016-06-25

**Authors:** Song Thi Le, Chisa Yasuoka, Haruyasu Asahara, Nagatoshi Nishiwaki

**Affiliations:** 1 Faculty of Applied Sciences, Ton Duc Thang University, 19 Nguyen Huu Tho Street, Tan Phong Ward, District 7, Ho Chi Minh City 700000, Vietnam; lethisong@tdt.edu.vn; 2 School of Environmental Science and Technology, Kochi University of Technology, Tosayamada, Kami, Kochi 782-8502, Japan; chorogi.max.x@gmail.com; 3 Research Center for Material Science and Engineering, Kochi University of Technology, Tosayamada, Kami, Kochi 782-8502, Japan

**Keywords:** 2-methylquinoline, iodine-mediated reaction, aniline, vinyl ether, redox reaction

## Abstract

A metal-free, mild and efficient method for the synthesis of 2-methylquinolines was successfully developed by condensation of anilines with vinyl ethers in the presence of catalytic amount of iodine. Modification of both pyridine and benzene moieties was easily achieved by changing only the vinyl ether and aniline. In this reaction, the iodine species was revealed to show dual behavior; molecular iodine serves as an oxidant, while its reduced form, hydrogen iodide, activates the vinyl ether. The redox reaction between these iodine species enables the use of a catalytic amount of iodine in this synthetic method.

## 1. Introduction

Iodine-catalyzed reactions have attracted much attention as environmentally sustainable alternatives to transition metal catalysis in industrial chemistry for producing commodity and specialty chemicals, foods, medicines, and pharmaceuticals [1]. Iodine undergoes oxidative addition, ligand exchange, reductive elimination, and ligand coupling, playing a role similar to that of transition metal catalysts [2]. In contrast to poisonous and expensive transition metals, molecular iodine is an environmentally friendly, inexpensive, and readily available reagent. The mild Lewis acidity of iodine also facilitates its use in organic synthesis, from stoichiometric levels to catalytic amounts. Thus, iodine-mediated reactions have been explored as a powerful method for the synthesis of many organic compounds [3,4,5,6,7,8]. 

Meanwhile, the importance and usefulness of quinoline derivatives has considerably increased in the pharmaceutical industry. Among them, 2-methylquinoline derivatives have versatile pharmacological properties such as antibacterial [9], antimalarial [10,11], anti-tumor [12] and anti-HIV [13,14] properties, and can act as nociceptin receptor antagonists [15]. 2-Methylquinolines can also be used as precursors in the synthesis of styrylquinolines, which are potential inhibitors of HIV-1 integrase and the replication of HIV-1 in cell culture [16]. Various quinolines for the treatment of protozoal and retroviral co-infection are also synthesized from 2-methylquinoline [17]. Furthermore, the 7-methoxy derivative is known to be a non-peptide bradykinin B_2_ receptor antagonist [18], and the 8-methoxy derivative is used as an anti-neurodegenerative agent, a radioprotective [19] and an antibiotic against *Staphylococcus aureus* [20]. In addition to the aforementioned pharmaceutical uses, substituted 2-methylquinolines are often employed as precursors for electronic and optoelectronic materials [21,22].

Despite their great importance, 2-methylquinolines are commonly prepared using traditional methods developed by Doebner-von Miller [23], Skraup [24], Conrad-Limpach-Knorr [25], Friedlaender [26] and Pfitzinger [27]. Unfortunately, these methods suffer from several disadvantages such as low yields due to side reactions; harsh reaction conditions, including the use of strong acids; multi-step reactions; and low regioselectivity. Although an acid-free approach using transition metal catalysts overcomes these disadvantages [28,29,30], an additional purification step is required to remove metal contaminants from the product. Furthermore, limited functional group tolerance diminishes the generality of this method [28,29,30]. Several researchers have reported synthetic methods using inexpensive Lewis acids such as iron(III) chloride, magnesium perchlorate, and zinc chloride [26,31,32,33,34,35,36]; however, these methods still have drawbacks, including the need for harsh reaction conditions, difficult work-up procedures, low yields, and high catalyst loadings.

Recently, an iodine-mediated reaction was applied to the synthesis of quinoline derivatives. Wang and co-workers reported a molecular iodine-catalyzed reaction for the synthesis of substituted quinolines from imines and aldehydes [37]. Furthermore, they reported the highly efficient multi-component synthesis of quinoline derivatives using catalytic amounts of molecular iodine [38,39,40,41]. Later, Wu et al. improved this method for the synthesis of quinolines and polycyclic quinolines [42]. Owing to the numerous advantages of molecular iodine over transition metals, an iodine-catalyzed quinoline synthesis was also reported in which anilines were condensed with cyclic vinyl ethers, such as 2,3-dihydrofuran and 3,4-dihydro-2*H*-pyran [43]. In this reaction, iodine was reported as serving only as a Lewis acid. In contrast, we report here a metal-free and efficient method for the synthesis of substituted 2-methylquinolines using low toxicity, low cost, commercially available iodine, anilines, and acyclic ethers under mild reaction conditions. We also suggest that the molecular iodine serves as an oxidant, which has not been proposed previously. In addition, a reaction mechanism is proposed, which includes the dual behavior of iodine species.

## 2. Results and Discussion

### 2.1. Iodine-Catalyzed Synthesis of 6-Substituted 2-Methylquinolines

Initially, the reaction of *p*-methoxyaniline **1a** with ethyl vinyl ether **2a** in dichloromethane was used as model reaction to optimize conditions (Table 1). The reaction did not proceed in the absence of iodine, with 88% of **1a** recovered (entry 1). On the other hand, quinoline **3a** [44] was successfully synthesized in 44% yield when the reaction was carried out in the presence of 5 mol % iodine, indicating that iodine was necessary for the construction of quinoline **3a** (entry 2). Among the three solvents tested, benzene was found to be the most suitable for this reaction (entries 2–4). While 5 mol % iodine was enough for the reaction to operate efficiently, the yield of quinoline **3a** decreased along with a 71% recovery of **1a**, when the catalyst loading was reduced to 1 mol % (entries 4−6). Consequently, the reaction conditions used in entry 4 were determined to be the optimal conditions.

With the optimized conditions in hand, we applied the reaction to the syntheses of 2-methylquinolines **3b**–**h** [29,45,46,47,48,49,50] using anilines **1b**–**h** (Table 2). The reaction was influenced by substituents on the benzene ring. When *m*-methoxyaniline **1b** and *o*-methoxyaniline **1c** were used, the reactions proceeded in a similar fashion, affording corresponding quinolines **3b** and **3c** in lower yields, presumably due to the electron-withdrawing inductive effect and steric hindrance of the methoxy group (entries 2 and 3). In contrast, aniline possessing both *o*- and *m*-methoxy groups (**1d**) had a higher product yield, indicating that high electron density on the benzene ring overcomes the aforementioned disadvantages (entry 4). In the case of aminophenol **1e**, no detectable **3e** was produced due to side reactions, such as the oxidation of **1e** (entry 5). Anilines connected to another electron-donating group, **1f** and **1g**, reacted efficiently to afford aminoquinoline **3f** and methylquinoline **3g**, respectively (entries 6 and 7). Unsubstituted aniline **1h** was also a suitable substrate in this reaction, but with somewhat lower efficiency.

To further demonstrate the scope of this reaction, various vinyl ethers, **2a**–**f**, were subjected to the reaction with *p*-methoxyaniline **1a** (Table 3). Interestingly, quinoline **3a** was produced despite the ether alkoxy group being changed from ethoxy to isobutoxy (entries 1 and 2), indicating that the pyridine moiety in quinolines **3** was derived from the vinyl group. Indeed, propenyl ether **2c** afforded 2-ethyl-3-methylquinoline **4** [51] in good yield, which is employed in the preparation of metal acid corrosion inhibitors, sorbents, and cyanine dyes [52]. When 2,3-dihydrofuran **2d** was used, a quinoline possessing acetal functions, **5**, was synthesized (See the Supplementary Materials). Desired quinolines **6** and **7** were not detected when electron-poor vinyl ethers **2e** and **2f** were employed (entries 5 and 6).


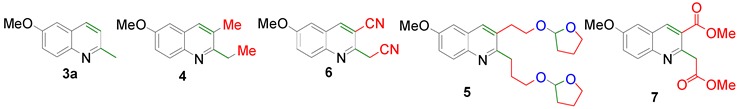


### 2.2. Study on the Mechanism 

**Scheme 1:**
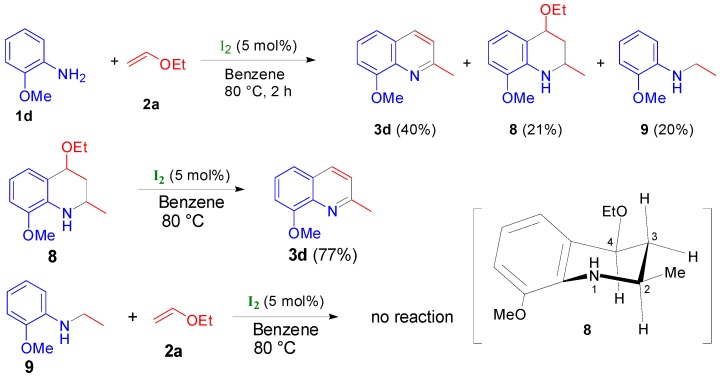
Reactions of by-products **8** and **9** with iodine under the same conditions.

In the reaction of *o*-methoxyaniline **1d** with vinyl ether **2a**, several products were formed. In order to gain insight into the mechanism, the reaction mixture was subjected to column chromatography and the products were identified as 4-ethoxy-1,2,3,4-tetrahydro-8-methoxy- 2-methylquinoline (**8**) as single diastereomer [53] and *N*-ethyl-2-methoxyaniline (**9**) [54] (Scheme 1). Although elucidation of the stereochemistry of compound **8** was attempted by ^1^H-NMR, it was not achieved due to two protons at the 3-position having the same coupling constant (12.0 Hz) with a proton at the 2-position. Considering steric repulsion, the stereochemistry was predicted to be the *cis*-isomer because the ethoxy and methyl groups are located at the equatorial position. Indeed, DFT calculation using B3LYP 6-31G(d,p) revealed that the *cis*-isomer was more stable than the *trans*-isomer, with 1.887 kcal/mol. Interestingly, tetrahydroquinoline **8** was converted to **3d** in 77% yield by heating in the presence of iodine. In contrast, no reaction was observed for **9** under the same conditions. These results suggest that **8** is an intermediate in the formation of **3d**, and that *N*-alkylaniline **9** is a by-product (Scheme 1).

**Figure 1:**
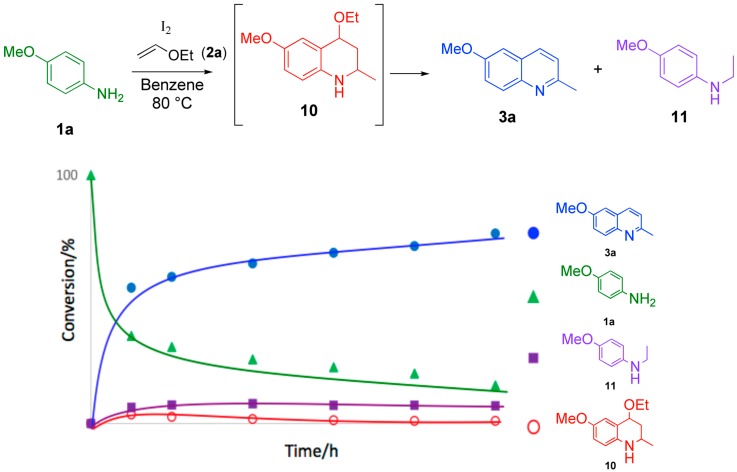
Monitoring the reaction of **1a** and **2a** by ^1^H-NMR.

The reaction of **1a** with vinyl ether **2a** was monitored by ^1^H-NMR (Figure 1). As starting material **1a** was consumed, product **3a** formed. Just after the reaction started, the formation of **10** [55] was observed, which gradually decreased over time. In addition, a small amount of **11** [54] was formed, but the amount was unchanged, even after 72 h. Thus, it was confirmed that **10** was a reaction intermediate and **11** was a by-product. 

**Scheme 2:**
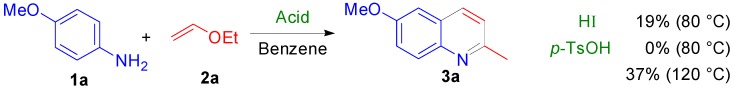
Synthesis of quinoline **3a** in the presence of acid catalyst.

In addition, hydriodic acid and *p*-toluenesulfonic acid were employed as catalysts in this reaction instead of iodine (Scheme 2). Hydriodic acid catalyzed this reaction, but gave a lower yield of **3a**. *p*-Toluenesulfonic acid did not catalyze the reaction at 80 °C, and it was necessary to heat at 120 °C. Although the reaction proceeded in the presence of an acid catalyst, iodine was necessary to accelerate the reaction smoothly under milder conditions. 

**Scheme 3:**
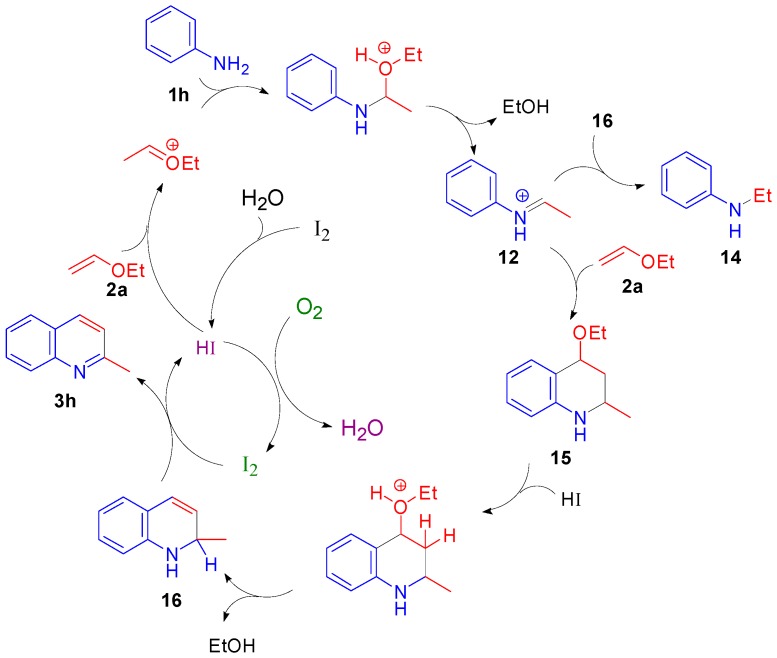
A plausible mechanism.

Based on these experimental results, the reaction mechanism in Scheme 3 was proposed. At first, iodine generates a trace amount of hydrogen iodide by reacting with water present in the reaction mixture [56], initiating the reaction. Aniline **1h** attacks the activated vinyl group of **2a**, leading to an *N,O*-acetal, from which ethanol is eliminated to afford iminium intermediate **12**. Iminium **12** is considered a common intermediate for both *N*-ethylaniline **14** and tetrahydroquinoline **15**. Tetrahydroquinoline **15** is formed by the attack of another molecule of **2a** to iminium ion **12** followed by intramolecular cyclization, while dihydroquinoline **16** is obtained as a result of ethanol elimination. Oxidation of **16** by iodine affords the final product, quinoline **3h**. In this process, iodine is reduced to hydrogen iodide, which then returns to iodine upon contact with air. Indeed, when the reaction was conducted under Ar atmosphere, the product was obtained in lower yield. Thus, oxygen may assist the reaction. Therefore, a catalytic amount of iodine is enough to produce this reaction. In contrast, when intermediate **12** is reduced by dihydroquinoline **16**, *N*-alkylaniline **14** is formed as a by-product.

## 3. Materials and Methods

### 3.1. General Information 

All the reagents and solvents were commercially available and used as received. The ^1^H-NMR spectra were measured on a Bruker Ascend-400 (Bruker, Billerica, MA, USA) at 400 MHz with TMS as an internal standard. The ^13^C-NMR spectra were measured on a Bruker Ascend-400 at 100 MHz, and assignments of ^13^C-NMR spectra were performed by DEPT experiments. The high resolution mass spectra were measured on a AB SCIEX Triple TOF 4600 (AB Sciex, Framingham, MA, USA). 

### 3.2. Procedures 

#### Iodine-Mediated Synthesis of 2-Methylquinolines **3**

To a solution of vinyl ether **2a** (192 µL, 2 mmol) in benzene (10 mL), were added *p*-methoxyaniline (**1a**, 123.2 mg, 1 mmol) and iodine (12.7 mg, 0.05 mmol), and the resultant mixture was heated at 80 °C for 2 h. The reaction mixture was washed with saturated sodium thiosulfate solution (1 × 10 mL) to remove unreacted iodine, and dried over magnesium sulfate. After removal of solvent, the residue was subjected to silica gel column chromatography (eluent: hexane/ethyl acetate = 95/5) to afford **3a** (110.7 mg, 0.64 mmol, 64%). 

The reactions of the aniline **1** with other vinyl ether **2** were performed in a similar manner.

### 3.3. Compound Characterizations 

*6-Methoxy-2-methylquinoline* (**3a**) [44]: ^1^H-NMR (400 MHz, CDCl_3_): δ 7.94 (d, *J* = 8.3 Hz,1H), 7.91(d, *J* = 9.1 Hz,1H), 7.33 (dd, *J* = 9.1, 2.8 Hz, 1H), 7.23 (d, *J* = 8.3 Hz, 1H), 7.04 (d, *J* = 2.8 Hz, 1H), 3.91 (s, 3H), 2.70 (s, 3H).

*7-Methoxy-2-methylquinoline* (**3b**) [45]: ^1^H-NMR (400 MHz, CDCl_3_): δ 7.95 (d, *J* = 8.2 Hz, 1H), 7.63 (d, *J* = 8.8 Hz, 1H), 7.37 (s, 1H), 7.14 (d, *J* = 8.2 Hz, 1H), 7.13 (d, *J* = 8.8 Hz, 1H), 3.93 (s, 3H), 2.71 (s, 3H).

*8-Methoxy-2-methylquinoline* (**3c**) [46]: ^1^H-NMR (400 MHz, CDCl_3_): δ 8.00 (d, *J* = 8.4 Hz, 1H), 7.38 (dd, *J* = 8.4, 8.1 Hz, 1H), 7.34 (d, *J* = 8.1 Hz, 1H), 7.31 (d, *J* = 8.4 Hz, 1H), 7.03 (d, *J* = 8.4 Hz, 1H), 4.08 (s, 3H), 2.80 (s, 3H).

*5,8-Dimethoxy-2-methylquinoline* (**3d**) [47]: ^1^H-NMR (400 MHz, DMSO): δ 7.45 (d, *J* = 8.8 Hz,1H), 7.09 (d, *J* = 8.8 Hz,1H), 6.87 (d, *J* = 6.8 Hz, 1H), 6.66 (d, *J* = 6.8 Hz, 1H), 3.90 (s, 3H), 3.75 (s, 3H), 2.68 (s, 3H).

*6-(N,N-dimethylamino)-2-methylquinoline* (**3f**) [49]: ^1^H-NMR (400 MHz, DMSO): δ 7.98 (d, *J* = 8.4 Hz, 1H), 7.76 (d, *J* = 9.2 Hz,1H), 7.37 (dd, *J* = 9.2, 2.8 Hz, 1H), 7.22 (d, *J* = 8.4 Hz, 1H), 6.88 (d, *J* = 2.8 Hz, 1H), 2.97 (s, 6H), 2.56 (s, 3H).

*2,6-Dimethylquinoline* (**3g**) [29]: ^1^H-NMR (400 MHz, CDCl_3_): δ 7.96 (d, *J* = 8.3 Hz, 1H), 7.95 (d, *J* = 8.3 Hz, 1H), 7.52 (s, 1H), 7.51 (d, *J* = 8.3 Hz, 1H), 7.24 (d, *J* = 8.3 Hz, 1H), 2.74 (s, 3H), 2.51 (s, 3H).

*2-Methylquinoline* (**3h**) [50]: ^1^H-NMR (400 MHz, CDCl_3_): δ 8.04 (d, *J* = 5.0 Hz, 1H), 8.02 (d, *J* = 5.0 Hz, 1H), 7.77 (d, *J* = 8.0 Hz, 1H), 7.68 (dd, *J* = 8.4, 7.0 Hz, 1H), 7.48 (dd, *J* = 8.0, 7.0 Hz, 1H), 7.28 (d, *J* = 8.4 Hz, 1H), 2.75 (s, 3H).

*2-Ethyl-6-methoxy-3-methylquinoline* (**4**) [27]: ^1^H-NMR (400 MHz, CDCl_3_): δ 7.91 (d, *J* = 9.2 Hz, 1H), 7.69 (s, 1H), 7.25 (dd, *J* = 9.2, 2.8 Hz, 1H), 6.94 (d, *J* = 2.8 Hz, 1H), 3.87 (s, 3H), 2.94 (q, *J* = 7.5 Hz, 2H), 2.43 (s, 3H), 1.35 (t, *J* = 7.5 Hz, 3H).

*Compound **5**:* ^1^H-NMR (400 MHz, CDCl_3_): δ 7.89 (d, *J* = 9.2 Hz, 1H), 7.81 (s, 1H), 7.27 (d, *J* = 9.2 Hz, 1H), 6.99 (s, 1H), 5.14 (t, *J* = 2.0 Hz, 2H), 4.01−3.93 (m, 1H), 3.91 (s, 3H), 3.88−3.67 (m, 6H), 3.54−3.48 (m, 1H), 3.07−3.00 (m, 4H), 2.13−2.06 (m, 2H), 2.03−1.78 (m, 8H); ^13^C-NMR (100 MHz, CDCl_3_) 23.4 (CH_2_), 23.5 (CH_2_), 29.3 (CH_2_), 32.1 (CH_2_), 32.3 (two CH_2_ signals overlapped), 32.4 (CH_2_), 55.4 (CH_3_), 66.8 (four CH_2_ signals overlapped), 103.8 (CH), 103.9(CH), 104.7 (CH), 121.0 (CH), 127.9 (C), 130.0 (CH), 130.9 (C), 134.6 (CH), 142.8 (C), 157.2 (C), 158.9 (C); HRMS Calcd for C_23_H_31_NO_5_: 402.2275. Found: 402.2272. 

*Cis-4-Ethoxy-1,2,3,4-tetrahydro-8-methoxy-2-methylquinoline* (**8**) [29]: ^1^H-NMR (400 MHz, CDCl_3_): δ 7.00 (dd, *J* = 4.8, 4.8 Hz, 1H), 6.64 (d, *J* = 4.8 Hz, 2H), 4.70 (dd, *J* = 10.4, 5.6 Hz, 1H), 4.1 (br, 1H), 3.82 (s, 3H), 3.69–3.55 (m, 1H), 3.51 (dq, *J* = 9.2, 7.2 Hz, 2H), 2.21 (ddd, *J* = 12.0, 5.6, 2.4 Hz, 1H), 1.70 (ddd, *J* = 12.0, 10.4, 2.4 Hz, 1H), 1.28 (d, *J* = 7.0 Hz, 3H), 1.27 (dd, *J* = 7.2, 7.2 Hz, 3H).

*N-Ethyl-2-methoxyaniline* (**9**) [30]: ^1^H-NMR (400 MHz, CDCl_3_): δ 6.87 (dd, *J* = 7.6, 7.6 Hz, 1H), 6.76 (d, *J* = 7.6 Hz, 1H), 6.65 (dd, *J* = 7.6, 7.6 Hz, 1H), 6.60 (d, *J* = 7.6 Hz, 1H), 4.08 (br s, 1H), 3.84 (s, 3H), 3.16 (q, *J* = 7.2 Hz, 2H), 1.28 (t, *J* = 7.2 Hz, 3H).

## 4. Conclusions 

In conclusion, we have successfully developed an environmentally benign and efficient method for the construction of quinolines **3** from substituted anilines **1** and vinyl ethers **2** in the presence of an iodine catalyst. This protocol can be performed with simple manipulations in one step under mild conditions for both the reaction and work-up. Furthermore, no transition metals are used, which eliminates the need for a product decontamination step, thus considerably reducing the cost. Hence, this is a new synthetic method for quinoline derivatives that can be applied in various fields.

Furthermore, the roles of iodine species were also studied, finding that the iodine species had dual behavior, with molecular iodine serving as an oxidant, and its reduced form, hydrogen iodide, activating the vinyl ether. The redox reaction between these iodine species enables the use of a catalytic amount of iodine in this reaction.

## Figures and Tables

**Table 1:**
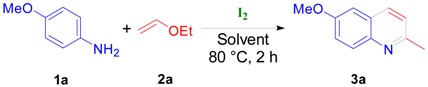
Synthesis of 6-methoxy-2-methylquinoline **3a**.

Entry	I_2_/mol %	Solv.	Yield/%	Recovery of 1a/%
1	0	CH_2_Cl_2_	0	88
2	5	CH_2_Cl_2_	44	22
3	5	MeCN	43	24
4	5	PhH	64	3
5	10	PhH	55	11
6	1	PhH	11	71

**Table 2:**
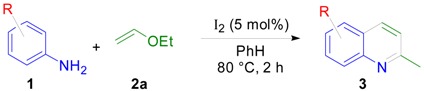
Modification of the benzene ring of quinoline **3**.

Entry	Aniline	Product	Yield of 3/%	Recovery of 1/%
1	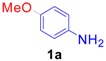	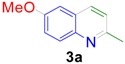	64	3
2	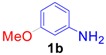	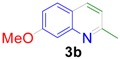	30	30
3	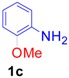	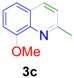	36	0
4	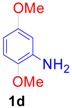	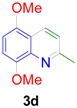	55	12
5	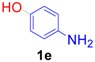	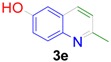	0	0
6	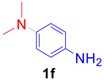	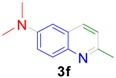	83	0
7	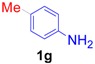	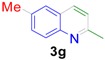	53	18
8	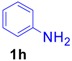	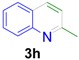	37	7

**Table 3:**
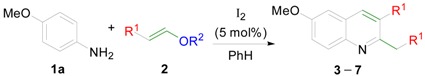
Modification of the pyridine ring of quinoline **3**.

Entry	Temp./°C	Time/h	Vinyl Ether	Product	Yield/%
1	80	2	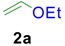	3a	64 ^1^
2	80	2	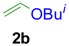	3a	54 ^1^
3	120	14	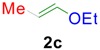	4	64
4	120	2	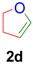	5	45
5	120	2	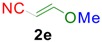	6	0
6	120	14	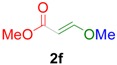	7	0

^1^ Recovery of **1a**: Entry 1 (3%), Entry 2 (33%).

## Supplementary Materials

Supplementary materials can be accessed at: http://www.mdpi.com/1420-3049/21/7/827/s1.
