# Supplementary material for: Dual Behavior of Iodine Species in Condensation of Anilines and Vinyl Ethers Affording 2-Methylquinolines
Source: Molecules. 2016 Jun 25;21(7):827. doi: 10.3390/molecules21070827 (PMC6272995; doi:10.3390/molecules21070827)
Supplement: Supplementary file 1 [file molecules-21-00827-s001.pdf]

Supplementary Materials: Dual Behavior of Iodine Species in Condensation of Anilines and Vinyl Ethers Affording 2-Methylquinolines

Song Thi Le, Chisa Yasuoka, Haruyasu Asahara and Nagatoshi Nishiwaki

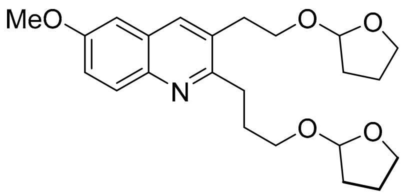

Compound 5

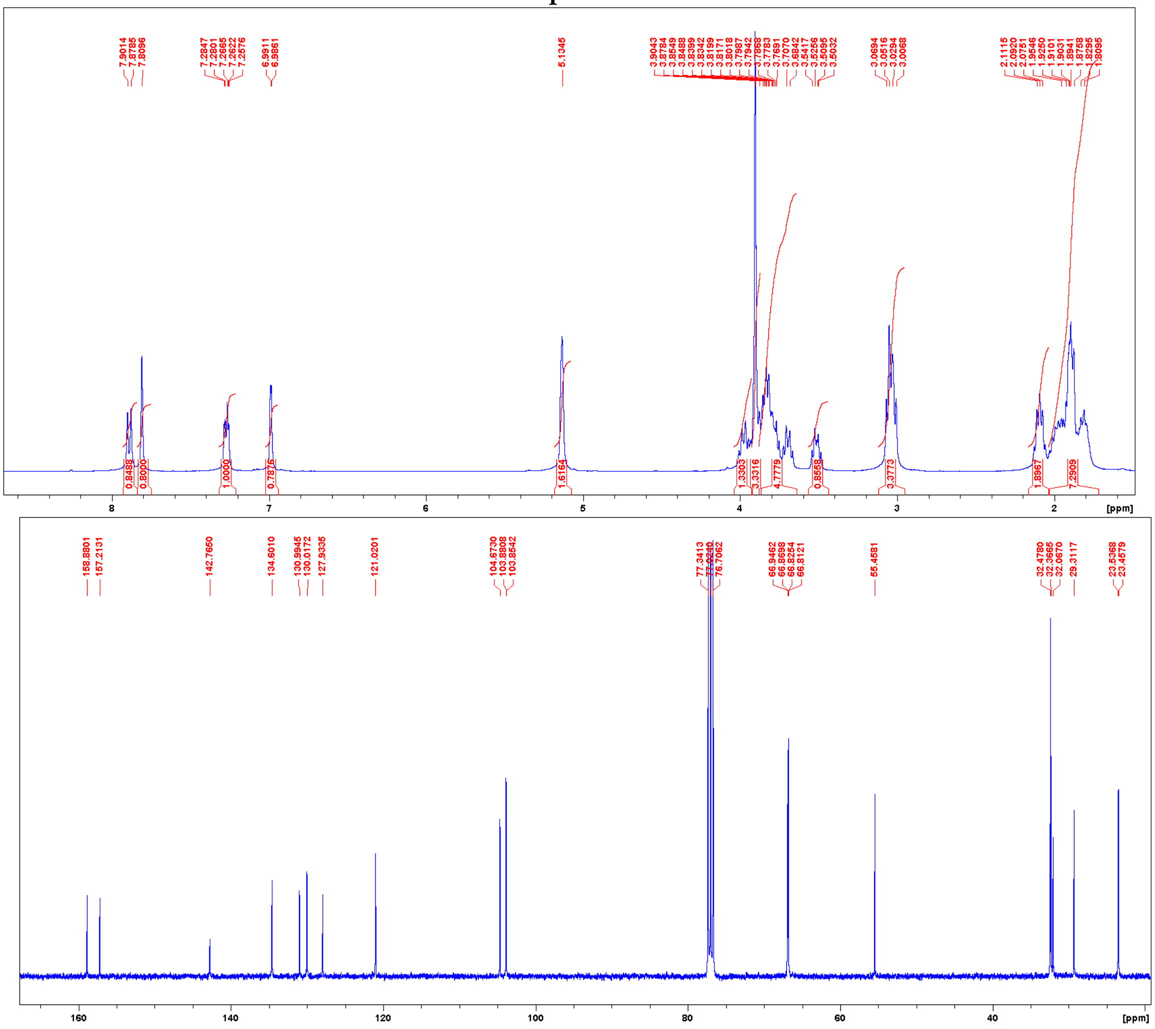

Figure S1. ^{1}H- and ^{13}C-NMR spectra of compound **5.**
